# Structural dynamics and stability of corticocortical and thalamocortical axon terminals during motor learning

**DOI:** 10.1371/journal.pone.0234930

**Published:** 2020-06-19

**Authors:** Ryota Hasegawa, Teppei Ebina, Yasuhiro R. Tanaka, Kenta Kobayashi, Masanori Matsuzaki

**Affiliations:** 1 Division of Brain Circuits, National Institute for Basic Biology, Myodaiji, Okazaki, Japan; 2 Division of Behavioral Neurobiology, National Institute for Basic Biology, Myodaiji, Okazaki, Japan; 3 Department of Basic Biology, SOKENDAI (Graduate University for Advanced Studies), Okazaki, Japan; 4 Department of Physiology, Graduate School of Medicine, University of Tokyo, Tokyo, Japan; 5 Brain Science Institute, Tamagawa University, Tokyo, Japan; 6 Section of Viral Vector Development, National Institute for Physiological Sciences, Okazaki, Aichi, Japan; 7 Brain Functional Dynamics Collaboration Laboratory, RIKEN Center for Brain Science, Saitama, Japan; 8 International Research Center for Neurointelligence (WPI-IRCN), University of Tokyo Institutes for Advanced Study, Tokyo, Japan; Technical University Braunschweig, Zoological Institute, GERMANY

## Abstract

Synaptic plasticity is the cellular basis of learning and memory. When animals learn a novel motor skill, synaptic modifications are induced in the primary motor cortex (M1), and new postsynaptic dendritic spines relevant to motor memory are formed in the early stage of learning. However, it is poorly understood how presynaptic axonal boutons are formed, eliminated, and maintained during motor learning, and whether long-range corticocortical and thalamocortical axonal boutons show distinct structural changes during learning. In this study, we conducted two-photon imaging of presynaptic boutons of long-range axons in layer 1 (L1) of the mouse M1 during the 7-day learning of an accelerating rotarod task. The training-period-averaged rate of formation of boutons on axons projecting from the secondary motor cortical area increased, while the average rate of elimination of those from the motor thalamus (thalamic boutons) decreased. In particular, the elimination rate of thalamic boutons during days 4–7 was lower than that in untrained mice, and the fraction of pre-existing thalamic boutons that survived until day 7 was higher than that in untrained mice. Our results suggest that the late stabilization of thalamic boutons in M1 contributes to motor skill learning.

## Introduction

Synapses consist of presynaptic and postsynaptic sites, and are indispensable prerequisites for communication between neurons. Functionally, synapses can change the strength of their chemical transmission by processes such as modification of the probability of transmitter release and varying the number of postsynaptic glutamate receptors [1, 2]. Anatomically, synapses can be newly formed, eliminated, or undergo a change in size [3–5]. Such functional and/or structural changes are termed synaptic plasticity, and form the cellular basis of learning and memory. In the majority of excitatory synapses in the mammalian brain, axonal boutons contact with dendritic spines [6], and the size of the dendritic spines correlates strongly with the excitatory postsynaptic response [7, 8]. Many spines are maintained for a long period of time and probably play critical roles in lifelong memory storage and the stability of neural circuits. By contrast, a subset of spines are dynamic; they can be newly formed, eliminated, or changed in size, and such structural plasticity probably plays critical roles in memory formation and extinction, homeostasis, and reorganization of neural circuits [3–5]. In fact, motor learning induces synaptic plasticity in M1 [9–11]. *In vivo* two-photon imaging in rodents revealed that dendritic spines in M1 dynamically change during motor learning [12–16], and that the stabilization of a subset of L1 spines of layer 5 (L5) pyramidal neurons, which are newly formed in the early stage of learning, is relevant to improvement in motor performance [14–16]. The erasure of these newly formed and/or enlarged spines causes impairment of the learned motor performance [17].

However, the structural plasticity of axonal boutons in M1 during motor learning is poorly understood, even though two-photon microscopy has been used to image axonal boutons *in vivo* [18–22]. Electron microscopy studies revealed that newly formed spines in the barrel cortex frequently make synapses with pre-existing axonal boutons [23], and that the dynamics of axonal boutons may therefore be different from those of dendritic spines. In addition, L1 dendritic spines in M1 receive not only axons from M1 neurons, but also axons from neurons in the secondary motor cortex (M2) and motor thalamus [24–29], with M2 and the motor thalamus being necessary for motor skill learning [27, 30, 31]. M2 neurons also project their axons to the spinal cord, and are involved in distal forelimb movement [32]. M2 inputs mainly excite L5 neurons in M1, whereas motor thalamic inputs excite M1 neurons in both layer 2/3 (L2/3) and L5 [29, 33, 34], with the thalamocortical synapses in M1 showing functional plasticity [35, 36]. The motor thalamus transmits signals from the basal ganglia and cerebellum, both of which are crucial for motor learning [37], to M1 [27]. Therefore, we hypothesized that the M2–M1 and thalamocortical pathways have distinct roles in motor learning, and that their axonal boutons would show distinct structural plasticity. In this study, we separately labeled long-range axons originating from M2 and the thalamus, and used two-photon microscopy to pursue the structural plasticity of these boutons in M1 during learning of an accelerating rotarod task. We found that the formation rate of M2 boutons was high during learning, whereas thalamic boutons showed a slight increase in their sizes in the early stage of learning and a decrease in their elimination rate in the late stage.

## Materials and methods

### Animals

All animal experiments were approved by the Animal Experimental Committee of the University of Tokyo Graduate School of Medicine (P16-012), and were performed in accordance with the institutional guidelines for the care and use of experimental animals. Two-month-old C57BL/6J male mice were purchased from SLC (Shizuoka, Japan). All mice were provided with food and water *ad libitum* in a 12 h:12 h light:dark cycle (lights on from 7 a.m. to 7 p.m.), and were not used for other experiments before the present study.

### Virus production

AAV1-CaMKII-Cre was obtained from the University of Pennsylvania Viral Vector Core (Cat#AV-1-PV2396). AAV1-CAG-FLEX-GFP and AAV1-CAG-FLEX-tdTomato were obtained from Addgene (#51502-AAV1, #51503-AAV1). pAAV-FLEX-GFP (Addgene #28304) and pAAV-FLEX-tdTomato (Addgene #28306) were gifts from Edward Boyden (Massachusetts Institute of Technology). AAVDJ/8-CAG-FLEX-tdTomato and AAVDJ/8-CAG-FLEX-GFP were produced with pAAV2-1 and purified as described previously [38, 39].

### Animal surgery for *in vivo* imaging

Virus injections and craniotomy were performed as described previously [27, 40]. Dexamethasone (2 mg/kg, intramuscularly), sulfadiazine (24 mg/kg, intraperitoneally), trimethoprim (4.8 mg/kg, intraperitoneally), and carprofen (6 mg/kg, intraperitoneally) were administered for preoperative treatment. To label axonal boutons with fluorescent protein, 0.10–0.15 μl of a 1:1 mixture of diluted adeno-associated virus (AAV) expressing Cre (AAV1-CaMKII-Cre, 1.6 × 10^9^ vg/ml), and either Cre-dependent AAV vector encoding GFP (AAVDJ/8-CAG-FLEX-GFP, 3.7 × 10^12^ vg/ml; AAV1-CAG-FLEX-GFP, 1.0 × 10^13^ vg/ml) or tdTomato (AAVDJ/8-CAG-FLEX-tdTomato, 7.0 × 10^12^ vg/mL; AAV1-CAG-FLEX-tdTomato, 6.3 × 10^12^ vg/ml) was stereotaxically injected into the left M2 region that overlapped with the rostral forelimb area (2.1 mm anterior and 0.7 mm lateral to bregma, and 0.3 mm below the brain surface) and/or the motor thalamus (1.07 mm posterior and 1.1 mm lateral to bregma, and 3.6 mm below the brain surface). Following the procedure, the small skull opening was covered with Kwik-Sil (World Precision Instruments, FL). Craniotomy surgery was subsequently performed above the left M1 caudal forelimb area (0.5 mm anterior and 1.5 mm lateral to the bregma) [24, 41–43], and the exposed dura was covered with a coverslip (2 mm diameter; 0.13–0.17 mm thickness), which was attached to a stainless metal ring (2.0 mm outer diameter; 1.9 mm inner diameter; 0.5 mm length) with optical adhesive (NOA81; Norland Products, NJ). The space between the brain surface and skull was filled with 1.2% agarose L (Wako, Osaka, Japan) and then sealed with cyanoacrylate (Vetbond; 3M, MN) and resin cement (Super Bond; Sun Medical, Shiga, Japan). A small stainless-steel plate (2 × 15 mm, 1 mm thick) was subsequently attached to the right hemisphere using resin cement (Estecem II; Tokuyama Dental, Tokyo, Japan). All surgical procedures were performed under anesthesia with an intraperitoneal injection of ketamine (74 mg/kg) and xylazine (10 mg/kg), and additional isoflurane inhalation (0.4–1%) as necessary. After surgery, the mice were administered a carprofen-containing jelly (MediGel CPF; Clear H_2_O, ME) for at least 3 days for inflammation management. To allow for sufficient protein expression, rotarod training and in vivo imaging were commenced 2–3 weeks after virus injection.

### Histology

To retrogradely label the neurons projecting to L1 in M1, a filter paper immersed with fluorochrome Fast Blue (Polysciences, 17740, PA) was placed on the brain surface of the caudal forelimb area of M1 (approximately within 0–1 mm anterior and 1–1.5 mm left of the bregma; n = 3 mice), as described previously [26, 27]. After 5–7 days, the mice were deeply anesthetized with ketamine (74 mg/kg) and xylazine (10mg/kg), and then transcardially perfused with PBS (10 ml) followed by 4% formaldehyde (30 ml). Their brains were postfixed with the same fixative overnight at 4°C and were then coronally sectioned (50 μm). Some sections were counterstained with NeuroTrace green (1:500; Invitrogen, CA; n = 3 mice), and some were incubated with mouse monoclonal anti-GAD67 (1:200, MAB5406; Millipore, MA; n = 2 mice) followed by goat anti-mouse IgG (Alexa Fluor-647, 1:200; Invitrogen).

All virus injection sites were confirmed in 100 μm thick coronal sections counterstained with DAPI (1:1000; Sigma-Aldrich, MO) or NeuroTrace deep red (1:500; Invitrogen). Images were acquired and automatically tiled with a BZ-X700 fluorescence microscope (Keyence, Osaka, Japan).

### Rotarod training

The rotarod training was performed with a custom-made apparatus [44] according to previously described procedures [14, 16]. A nylon polyamide knurling rod with a diameter of 30 mm and length of 60 mm was used. The rod had a 0.5-mm deep ridge to help prevent the mice from slipping. Both ends of the rod were supported by 300-mm diameter acrylate panels at a height of 300 mm above the base of the apparatus. Rotarod training was performed in the late afternoon (4 p.m. to 7 p.m.), which was near to the time reported to be appropriate for motor learning (8 p.m.) [14]. Before each day’s training, the mice were placed on the rod and left alone for 3 min to allow habituation to the rod. After this habituation, the rotation speed was linearly increased from 3 to 100 rotations per minute (RPM) over 3 min. The task performance was determined according to the rotation speed at which the mouse was unable to keep up. Twenty trials with 30 s intervals were performed each day over 7 consecutive days. In one mouse, the day-averaged rotarod speed to falling fluctuated, and did not increase from day 1 to day 7; this mouse was therefore removed from the analysis. The dynamics of dendritic spines in mice that perform motor tasks that do not require skilled learning do not differ from those in mice not performing such tasks [15]. Therefore, we used mice that were maintained in the home cage as control mice.

### *In vivo* two-photon imaging

Two-photon images were acquired with an FVMPE-RS system (Olympus, Tokyo, Japan) with a broadly tunable ultrafast laser (InSight DS-OL; Spectra-Physics, CA) tuned to 925 nm. The laser power under the objective was adjusted to be within the range of 2–10 mW. Fluorescence emission was collected with a 25× objective with a numerical aperture of 1.05 (XLPLN25XWMP, Olympus), separated with a 570 nm dichroic mirror (Olympus) and bandpass filters (495–540 nm for GFP; 575–630 nm for tdTomato), and detected with a GaAsP photomultiplier tube (PMT; Hamamatsu Photonics, Shizuoka, Japan).

For chronic imaging, mice were anesthetized with approximately 70% of the surgical dose of ketamine and xylazine under the two-photon microscope, with the application of eye ointment. In both trained and control mice, imaging was conducted from 7 p.m.–10 p.m. In the first imaging session, a bright-field image of the cortical surface of the imaging areas was obtained to allow the vascular patterns to be used as landmarks on the following imaging days. In each imaging session, the laser power and PMT voltage were adjusted to make the fluorescent signals of axons as constant as possible over all sessions. On days 2, 4, and 7 the imaging in the trained mice was started 1–3 h after the end of training, because a previous study revealed that learning-related spine formation was observed within 1 h of the first training [15]. Two to seven fields were imaged per imaging session.

High resolution image stacks (1024 × 1024 pixels; 0.11 μm/pixel; 0.7 μm step along the optical axis) were obtained from L1 in M1 (number of imaging planes per field: M2 boutons in control mice, 30.1 ± 0.93 [mean ± SEM]; M2 boutons in trained mice, 28.0 ± 0.96; thalamic boutons in control mice, 29.9 ± 0.94; thalamic boutons in trained mice, 29.3 ± 0.97), with the imaged axons being located within 100 μm from the brain surface (depth of the imaging planes: M2 boutons in control mice, 30.9 ± 1.83 μm [mean ± SEM]; M2 boutons in trained mice, 29.2 ± 1.85 μm; thalamic boutons in control mice, 28.5 ± 1.59 μm; thalamic boutons in trained mice, 28.8 ± 1.85 μm). The lengths of traced axons per field were 238.2 ± 22.4 μm for M2 boutons in control mice (40 fields); 197.9 ± 9.1 μm for M2 boutons in trained mice (39 fields); 235.9 ± 11.1 μm for thalamic boutons in control mice (32 fields); and 213.4 ± 13.4 μm for thalamic boutons in trained mice (31 fields). Median filtering, projection and contrast enhancement were performed on representative images used only for visualization purposes.

### Image analysis

All image analysis was performed using Fiji [45] and custom-written MATLAB (Mathworks, MA) code. The method developed by Gala et al. [46] was used to calculate bouton size. Axons were carefully traced with Simple Neurite Tracer [47], and axons that were successfully traced over all imaging sessions were analyzed. Putative boutons were detected by BoutonAnalyzer [46], and their ‘bouton weight’, the normalized bouton size (bouton intensity divided by the Gaussian filtered intensity of the axonal shaft), was defined as the bouton size. Gala et al. reported that the smallest size of boutons with postsynaptic densities was 1.98; therefore, we defined putative boutons with a size >2 as boutons. Gala et al. also reported that three out of four putative boutons with a size of 1.98–2.85 were not coupled with postsynaptic densities. Therefore, the boutons used for the analyses shown in Figs 3–5 had a size >3 in at least one of the imaging sessions.

Boutons were considered identical between sessions if the difference in their distance from a visually identified adjacent landmark bouton was less than 0.7 μm. A newly formed bouton was defined as a bouton that appeared on day 2 or later in a position where boutons were not observed in the immediately preceding imaging days. An eliminated bouton was defined as a bouton that was observed in an earlier session but had since fallen in size to below 1.3 [18]. The rates of formation and elimination were respectively defined as the number of newly formed or eliminated boutons divided by the total number of boutons in the previous session. The turnover rate was defined as the average of the formation and elimination rates. The survival fraction of a population of boutons in each imaging session was defined as the number of identical boutons that survived in the session divided by the number of boutons in the initial imaging session. Boutons that disappeared and then reappeared at the same location (within 0.7 μm from the expected position) were considered to be identical boutons.

### Experimental design and statistical analysis

The Kolmogorov–Smirnov test was used for comparisons of bouton size distributions. Other statistical comparisons were performed using Student’s *t*-test. Correlation coefficients were calculated using Pearson’s correlation. All data are presented as mean ± SEM.

## Results

### M1 superficial layer receives synaptic inputs from M2 and thalamus

**Fig 1:**
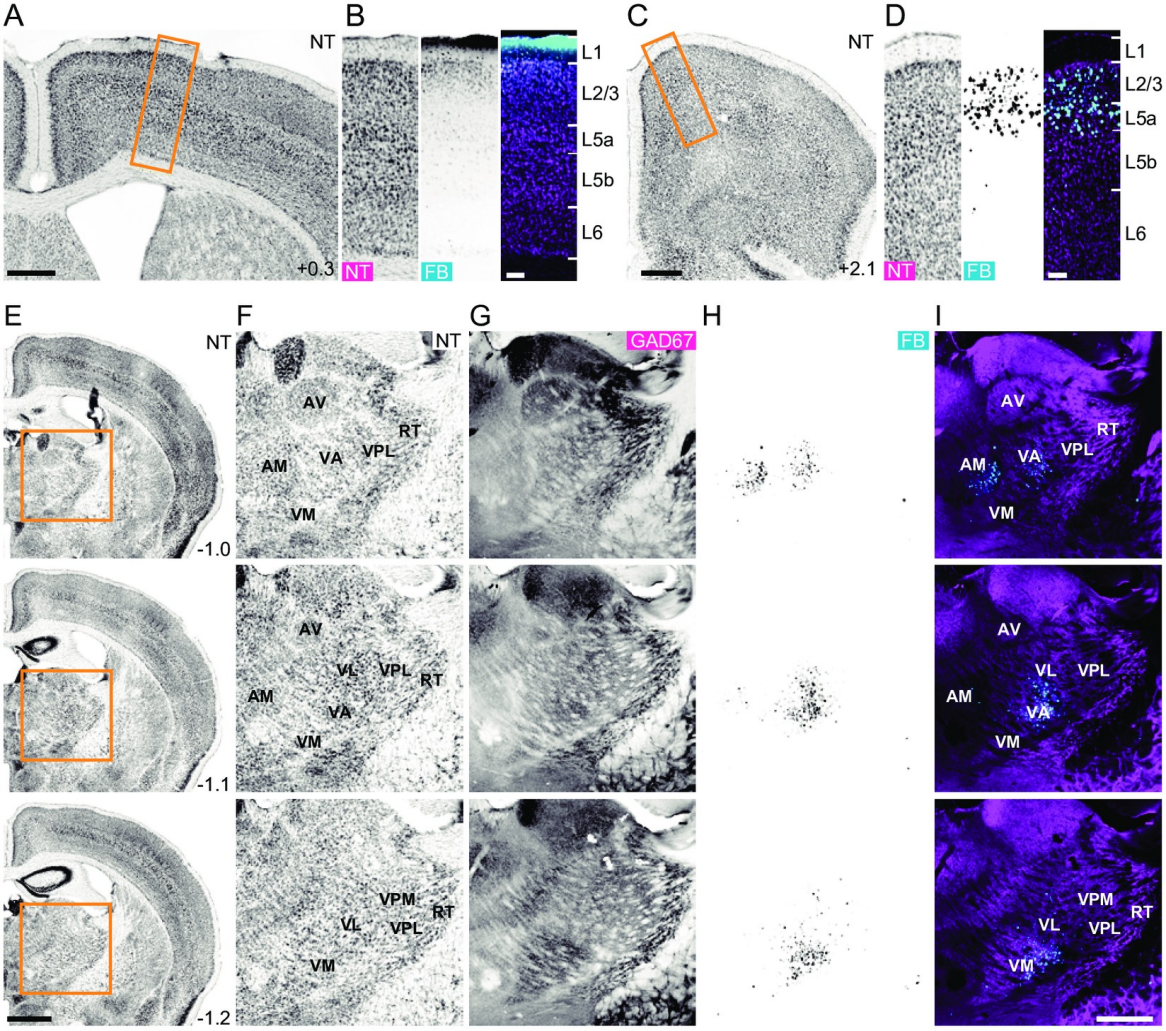
L1 in M1 receives axonal inputs from M2 and the motor thalamus. (A) NeuroTrace (NT) fluorescence in a coronal section including the Fast Blue (FB) immersion site in M1. Scale bar, 500 μm. (B) NT (left) and FB fluorescence (middle), and overlay image (colored magenta for NT and cyan for FB; right) of the boxed area in (A). Scale bar, 100 μm. (C) NT fluorescence in M2. Scale bar, 500 μm. (D) NT (left) and FB (middle) fluorescence, and overlay image (colored in magenta for NT and cyan for FB; right) of the boxed area in (C). Scale bar, 100 μm. (E) NT fluorescence in coronal sections including the motor thalamus. Scale bar, 1 mm. (F–H) Fluorescence images of NT (F), immunohistochemically stained GAD67 (G), and FB (H) in the boxed area in (E). (I) Overlay image of (G; magenta) and (H; cyan). Scale bar, 500 μm. The anteroposterior distance (mm) from the bregma is shown in the lower right corner of (A), (C), and (E). AM, anteromedial nucleus; AV, anteroventral nucleus; VA, ventral anterior nucleus; VL, ventral lateral nucleus; VM, ventral medial nucleus; VPL, ventral posterolateral nucleus; VPM, ventral posteromedial nucleus; RT, reticular nucleus.

First, we anatomically confirmed that L1 in M1 receives axonal projections from M2 and the thalamus. We placed a filter paper that had been immersed in the retrograde tracer Fast Blue onto the M1 surface for 3 min (Fig 1A and 1B). After 5–7 days, we detected fluorescence in M2 (Fig 1C and 1D) [28] and the thalamus, including the motor thalamic nuclei that mainly receive GABAergic inputs from the basal ganglia (the ventral anterior and ventral medial nuclei; Fig 1E–1I) [25, 27, 48, 49]. The anteromedial nucleus, which mainly receives projections from the medial mammillary nucleus, prelimbic, infralimbic, and anterior cingulate cortices [50, 51], was also labeled with the tracer. Given that both M2 and the motor thalamus send synaptic inputs onto apical tuft dendrites of L5 corticospinal neurons [29], we hypothesized that these brain areas could be candidates for the presynaptic counterpart of the newly formed learning-related spines of L5 pyramidal neurons that have been reported in previous studies [15, 16].

### Chronic *in vivo* two-photon imaging of long-range axonal boutons

**Fig 2:**
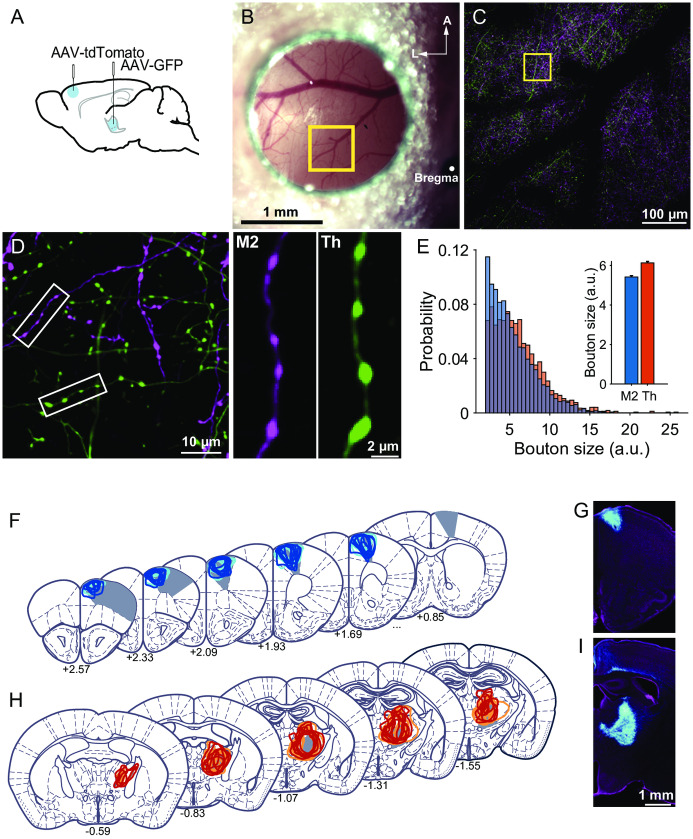
Two-photon imaging of M2 boutons and thalamic boutons. (A) Schematic illustration of the AAV injection. (B) Representative image of the M1 cranial window on the mice in which tdTomato was expressed in M2 neurons and GFP was expressed in thalamic neurons. A, anterior; L, lateral. (C) Maximum intensity projection of the two-photon images of the boxed area in (B). The images were acquired at depths of 0–100 μm from the brain surface. (D) Left, two-photon image of the boxed area in (C). The depth of the imaging plane was 30 μm. Right, images of an axon originating from M2 (left) and a thalamocortical axon (Th; right). (E) Left, the distribution of the size of all imaged M2 (blue) and thalamic (orange) putative boutons on the first imaging day (day 0; M2 boutons: n = 1793 from 16 mice; thalamic boutons: n = 1702 from 15 mice). Right, the mean size of M2 and thalamic (Th) boutons. The data include both trained and untrained mice. p < 1 × 10^−10^ (Kolmogorov–Smirnov test). (F, H) Localization of AAV injection sites. The infected area in each mouse is shown as colored lines. Filled gray areas indicate M2 (F), or VA, VL, and VM (H), according to Paxinos and Franklin [55]. Anteroposterior distance (mm) from the bregma is indicated for each section. Blue represents trained mice with M2 injection, and cyan represents control mice with M2 injection; red represents trained mice with thalamic injection, and orange represents control mice with thalamic injection. (G, I) Representative images of AAV-infected neurons (cyan) in M2 (G) and the motor thalamus (I). NeuroTrace counterstaining is shown in magenta.

Next, we injected AAV encoding either the tdTomato gene or GFP into M2 or the motor thalamus (Fig 2A). After 2–3 weeks, we used two-photon microscopy to image the fluorescently labeled axons and their *en passant* boutons in L1 of M1 in anesthetized mice (Fig 2B–2D). The bouton size, which was estimated from the fluorescence intensity [46] (see Materials and methods for details), was larger in thalamic boutons than in M2 boutons (p = 2.65 × 10^−11^, Kolmogorov–Smirnov test; Fig 2E). This result is consistent with previous studies showing that thalamocortical synapses are larger than corticocortical synapses [52–54]. We confirmed that the imaged axons in M1 originated from M2 or the thalamus after the imaging experiments (Fig 2F–2I).

### The size of thalamic boutons was slightly increased in the early training period

**Fig 3:**
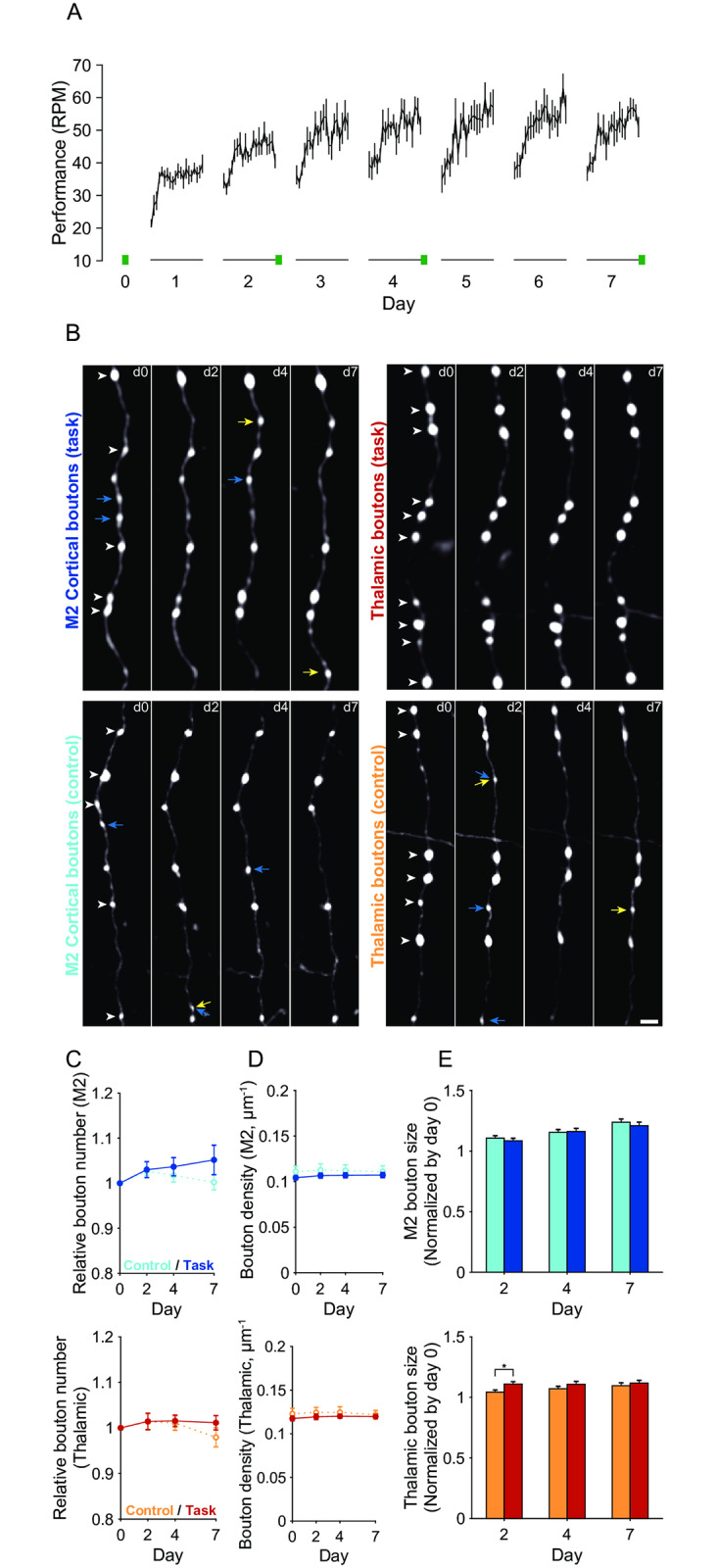
Two-photon imaging of the same M2 and thalamic boutons during motor learning. (A) Averaged motor performance over the 7 training days (n = 8). Performance is defined as the rotation speed at which the mice were unable to keep up (20 trials for each training day). Two-photon imaging was conducted on day 0 and on days 2, 4, and 7 after training (green bars). (B) Representative two-photon images of M2 (left) and thalamic (right) boutons in the trained (top) and control (bottom) mice on days 0, 2, 4, and 7. White arrowheads on day 0 images indicate the boutons that persisted from day 0 to day 7. Blue arrows indicate the boutons that were eliminated during the imaging sessions. Yellow arrows indicate the boutons that were newly formed during days 2–7. Scale bar, 2 μm. (C) Time course of the relative number of M2 (top) and thalamic (bottom) boutons in trained and control mice. For each animal, the number of boutons on each day was normalized to that on day 0. (D) Time course of the density of M2 (top) and thalamic (bottom) boutons in trained and control mice. (E) The relative bouton size of M2 (top) and thalamic (bottom) boutons in trained and control mice on days 2, 4, and 7. For each bouton, its size on each day was normalized to that on day 0. * p < 0.05.

Over a 7-day training period, a group of mice (n = 8 mice) were trained to perform an accelerating rotarod task with the rotation speed being increased from 3 to 100 RPM over 3 min. The average motor performance steadily increased over days 1–4, and then after day 4 the performance in the last trial on each day showed only a slight increase or no increase (Fig 3A). We pursued the axonal boutons in the same imaging fields under anesthesia on days 0 (the day immediately before the first training day), 2, 4, and 7 in both the trained mice (Fig 3A and 3B; 39 fields for M2 boutons in eight mice and 31 fields for thalamic boutons in seven mice) and untrained control mice (40 fields for M2 boutons in eight mice and 32 fields for thalamic boutons in eight mice). We estimated the size and number of boutons on each imaging day and found that the total numbers of M2 and thalamic boutons were similar between the trained and control mice across the imaging days (Fig 3C). The density of thalamic boutons was 0.12 ± 0.0035 μm^−1^ (Fig 3D), which was very similar to the density of L1 boutons on axons from the motor thalamus estimated using single-neuron tracing (0.11 ± 0.0011 μm^−1^) [25]. In the trained mice, the relative size of thalamic boutons on day 2 in comparison with their size on day 0 was slightly larger than in the control mice (day 2: *t*_(1411)_ = –2.57, p = 0.0104, unpaired *t*-test; Fig 3E). The sizes of thalamic boutons on day 7 and M2 boutons on days 2–7 were similar between the trained and control mice.

### M2 axonal boutons showed an increased rate of formation during motor learning

**Fig 4:**
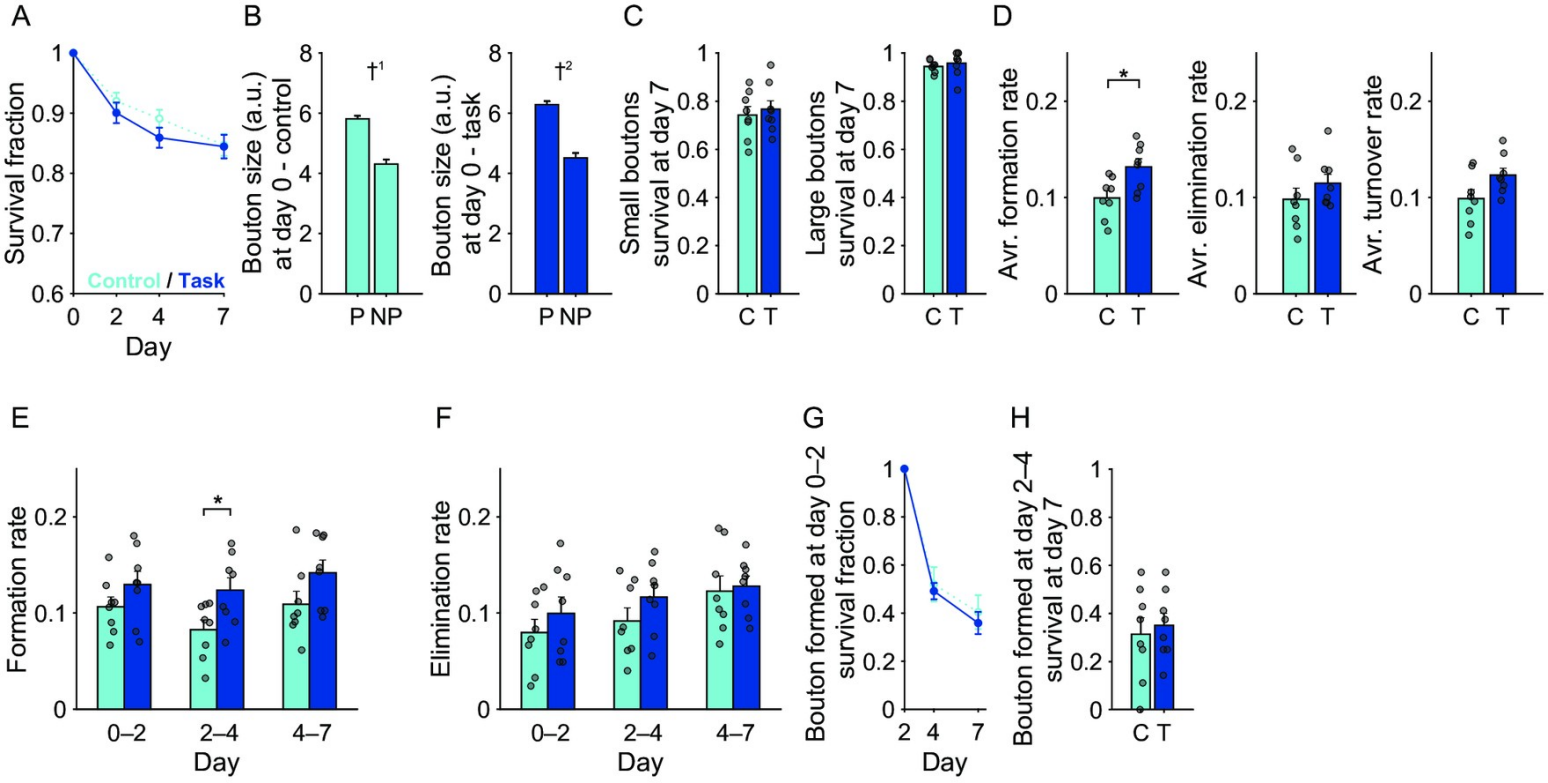
Dynamics and stabilization of M2 boutons during motor learning. (A) Survival fraction of the pre-existing M2 boutons up to day 7. Blue, M2 boutons in trained mice; cyan, M2 boutons in control mice. (B) The day 0 sizes of the pre-existing boutons that persisted until day 7 (P) and those that did not (NP). †^1^ p < 1 × 10^−8^, †^2^ p < 1 × 10^−9^. (C) Day 7 survival rates of small and large pre-existing boutons. Those pre-existing boutons whose sizes belonged to the lower and upper third were defined as small and large boutons, respectively. C, control mice; T, trained mice. (D) Averaged formation, elimination, and turnover rates over days 0–2, 2–4, and 4–7. * p < 0.05. (E) Formation rate of M2 boutons during days 0–2, 2–4, and 4–7. (F) Elimination rate of M2 boutons during days 0–2, 2–4, and 4–7. (G) Survival rate of the boutons that were newly formed during days 0–2. (H) Day 7 survival rates for those boutons that were newly formed during days 2–4. C, control mice; T, trained mice.

Next, we examined the stability and dynamics of M2 axonal boutons during learning. By examining the structure of the axonal arbors and the bouton locations in each imaging session, we determined which axonal boutons were consistent across imaging days and which boutons were newly formed or eliminated during days 0–2, 2–4, and 4–7. The survival rate of M2 boutons that existed on day 0 in both trained and control mice (pre-existing boutons) showed a gradual decrease over time (Fig 4A). The day 7 survival rates of pre-existing M2 boutons in trained and control mice were approximately 85%.

In both trained and control mice, the day 0 sizes of those pre-existing M2 boutons that were still present on day 7 were larger than those that were eliminated before day 7 (control mice, *t*_(828)_ = 6.05, p = 2.13 × 10^−9^; trained mice, *t*_(747)_ = 6.36, p = 3.49 × 10^−10^; unpaired *t*-test; Fig 4B). Thus, large boutons were more stable than small boutons. When the boutons were classified by their size on day 0 and the lower and upper thirds were defined as small and large, respectively, the day 7 survival rates of both small and large pre-existing thalamic boutons were not significantly different between trained and control mice (Fig 4C).

When the formation, elimination, and turnover rates (average of the formation and elimination rates) were averaged over days 0–2, 2–4, and 4–7, the formation rates of M2 boutons were higher in trained mice than in control mice (*t*_(14)_ = –2.83, p = 0.0134, unpaired *t*-test; Fig 4D). When the formation and elimination rates for each interval between imaging days were compared between the control and trained mice, only the formation rate during days 2–4 was significantly higher in trained mice than in control mice (*t*_(14)_ = –2.48, p = 0.0267, unpaired *t*-test; Fig 4E and 4F). These results suggest that formation of M2 boutons generally increased during the training days. This is consistent with the finding that the relative number of M2 boutons on day 7 in trained mice was slightly larger than that in control mice, although the difference was not statistically significant (Fig 3C). The survival rates of boutons that were newly formed over days 0–2 or 2–4 were similar between trained and control mice (Fig 4G and 4H).

### Thalamic axonal boutons showed a decreased rate of elimination during motor learning

**Fig 5:**
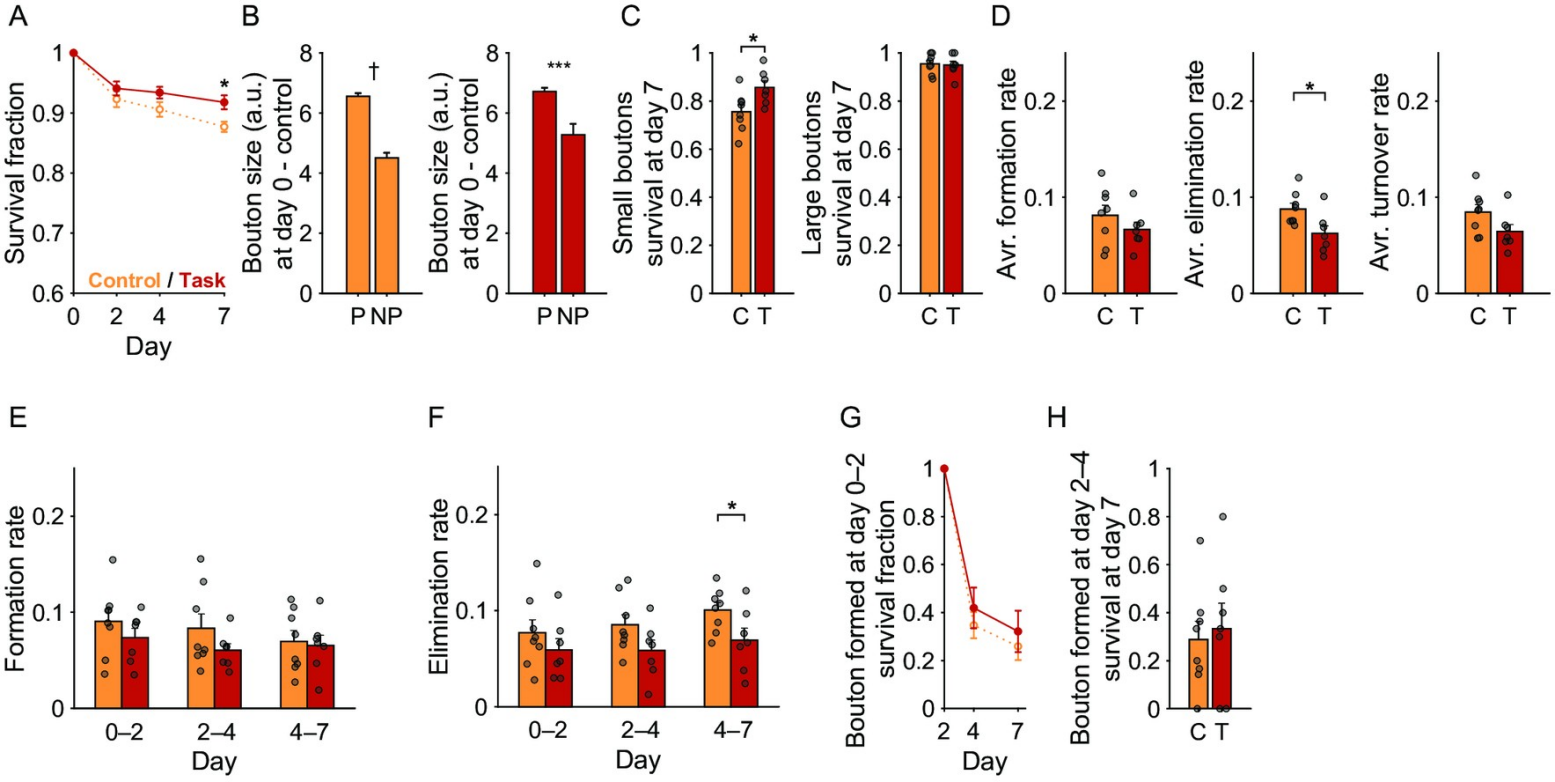
Dynamics and stabilization of thalamic boutons during motor learning. (A) Survival fraction of the pre-existing thalamic boutons up to day 7. Red, thalamic boutons in trained mice; orange, thalamic boutons in control mice. * p < 0.05. (B) The day 0 sizes of the pre-existing boutons that persisted until day 7 (P) and those that did not (NP). † p < 1× 10^−11^, *** p < 0.001. (C) Day 7 survival rates of small and large pre-existing boutons. Those pre-existing boutons whose sizes belonged to the lower and upper third were defined as small and large boutons, respectively. C, control mice; T, trained mice. (D) The formation, elimination, and turnover rates averaged over days 0–2, 2–4, and 4–7. (E) Formation rate of thalamic boutons during days 0–2, 2–4, and 4–7. (F) Elimination rate of thalamic boutons during days 0–2, 2–4, and 4–7. (G) Survival rate of the boutons that were newly formed during days 0–2. (H) Day 7 survival rates of those boutons that were newly formed during days 2–4. C, control mice; T, trained mice.

In contrast to M2 axonal boutons, the day 7 survival rate of pre-existing thalamic boutons was higher in trained mice than in control mice, exceeding 0.9 (*t*_(13)_ = −2.87, p = 0.0131, unpaired *t*-test; Fig 5A). Consistent with the M2 dynamics, the day 0 sizes of those pre-existing thalamic boutons that were still present on day 7 were larger than those that were eliminated before day 7 (control mice, *t*_(844)_ = 7.11, p = 2.50 × 10^−12^; trained mice, *t*_(714)_ = 3.35, p = 8.44 × 10^−4^; unpaired *t*-test; Fig 5B). By contrast, the day 7 survival rate of small pre-existing thalamic boutons was higher in trained mice than in control mice (*t*_(13)_ = −2.56, p = 0.0236, unpaired *t*-test; Fig 5C). These results suggest that some of the pre-existing thalamic boutons stabilized during motor learning.

In contrast to M2 axonal boutons, the training-period-averaged elimination rate of thalamic boutons was significantly lower in trained mice than in control mice (*t*_(13)_ = 2.58, p = 0.0229, unpaired *t*-test; Fig 5D), as was the elimination rate over days 4–7 (*t*_(13)_ = 2.18, p = 0.048, unpaired *t*-test; Fig 5E and 5F). This result was consistent with the finding that the day 7 survival rate of pre-existing thalamic boutons was higher in the trained mice (Fig 5A). These results suggest that the elimination of thalamic boutons generally decreased over the training days. The survival rates of boutons that were newly formed during days 0–2 or 2–4 were similar between the trained and control mice (Fig 5G and 5H).

## Discussion

In the current study, we examined the structural dynamics of boutons on long-range axons in L1 of M1 that originated either from M2 or the thalamus throughout a motor learning procedure. Imaging of AAV-transfected neurons through a cranial window might cause more damage to L1 tissue than transcranial imaging of transgenically labeled neurons [56]; however, it allowed us to detect relative differences in size between M2 and thalamic boutons without any apparent distortion in axonal arbors. The total number of boutons was stable during imaging sessions, and the density of thalamic boutons was similar to that estimated by another group [25]. Consistent with these findings, transcranial imaging of transgenically labeled neurons has shown axonal boutons in the barrel cortex to be relatively stable, even when mice are placed in a sensory-enriched environment [57]. Thus, we conclude that if any damage was induced by the current imaging method, it was not apparent on axonal morphology, and that the fluorescence signal was sufficiently strong and stable to detect some structural dynamics of axonal boutons across imaging sessions.

Using a similar rotarod learning task, Yang et al. [16] demonstrated that a proportion of L1 dendritic spines of L5 pyramidal neurons in M1 were newly formed during the first 2 days, whereas the current study showed that the size of thalamic boutons slightly increased over the same period. A previous correlative electron microscopy study revealed that newly formed spines frequently make synapses with pre-existing axonal boutons, and that such multisynapse boutons are generally large [23]. In addition, the volume of multisynapse boutons was correlated with the summed spine volume [58]. Taken together, a small subset of the pre-existing thalamic boutons, including the enlarged boutons, may make contact with newly formed spines during the early stage. Indeed, histological studies have shown that thalamocortical boutons are prone to forming multiple synapses [59–61]. When newly formed spines connect with pre-existing boutons, these multisynapse boutons are considered as transient [23, 62]. After the day 2 imaging, pruning of the newly formed and/or pre-existing spines from these boutons might occur, and therefore, the size increase in thalamic boutons was detected only on day 2.

Regardless of whether the thalamic boutons were pre-existing, newly formed, or large or small, their stabilization in the late stage of learning may contribute to the stabilization of newly formed spines and formation of the circuit relevant to the consolidation of motor performance. In the late stage of learning, task-relevant movement becomes more coordinated and automatic, and the dorsolateral striatum is strongly active [63, 64]. The motor thalamic neurons that project their axons to layer 1 of M1 receive projections from the internal segment of the globus pallidus and the substantia nigra pars reticulata [25, 27]. These areas are downstream of the dorsolateral striatum [65]; thus, the signals generated in the dorsolateral striatum might be transmitted from the motor thalamus to M1 through the stabilized axonal boutons and contribute to generation of the M1 activity that is required for the motor skill consolidation.

One limitation of our study is that we focused only on layer 1 of M1. A part of the motor thalamus (the ventral lateral nucleus) receives synaptic signals from the deep cerebellar nuclei. The cerebellum plays a critical role in body balance and posture maintenance, and is required for rotarod learning [66]. The ventral lateral nucleus projects mainly to deep layers of M1, but not to L1 [25, 27, 48]. These synaptic inputs are considered to cause larger responses in L2/3 and L5 postsynaptic neurons than in L1 inputs [25]. Tetanic stimulation of both the sensory cortex and ventral lateral nucleus induces long-term potentiation of thalamic inputs to layer 3 neurons in M1 [36]. Motor learning increases the thalamocortical synaptic responses in L5 corticospinal neurons in M1 [35]. These increases may mainly occur in thalamocortical synapses located in L2/3 and L5. Thus, axonal boutons in the deep layers may show more dynamic changes correlating with the extent of motor learning. Moreover, the plasticity of synapses between M1 neurons may also play an important role in motor skill learning. These possibilities should be examined in the future.

It is necessary to use electron microscopy to clarify whether newly formed spines make contact with pre-existing and/or newly formed thalamic boutons. Although axonal lengths and numbers of boutons have been reported for single motor thalamic neurons in layer 1 of the neocortex [25], the number of boutons in layer 1 of M1, or the number of thalamic neurons that project to layer 1 in M1, have not been counted. Thus, it remains difficult to estimate the number and density of thalamic boutons in layer 1 of M1, the proportion of layer 1 M1 neuron dendritic spines making contact with thalamocortical boutons, and which M1 neurons receive inputs from imaged axonal boutons. If presynaptic and postsynaptic sites could be simultaneously labeled [67], it would be possible to estimate the pathway-specific structural plasticity of axonal boutons and dendritic spines during learning, and to determine the proportion of newly formed spines that make contact with M2 and thalamic boutons.

Several studies revealed that axonal boutons exhibit plasticity during a novel experience. For example, auditory fear conditioning induces the formation of boutons on axons projecting from the lateral amygdala to L1 dendritic spines of L5 auditory cortical neurons [68], and monocular deprivation induces a small and slow formation rate increase and elimination rate decrease in the axonal boutons of L2/3 neurons in the visual cortex [69]. By contrast, the axonal boutons of L2/3 neurons are stable in the barrel cortex in adult mice in an enriched environment [57]. In addition, single axonal boutons occasionally have multiple postsynaptic sites [23]. The same axons can make synapses with both excitatory and inhibitory neurons across layers and across different brain areas. Thus, axonal boutons may possess a greater variety of structural dynamics than dendritic spines, although the changes are smaller and slower on average, and the dynamics may depend on the types of presynaptic and postsynaptic neurons, brain areas, experience, and learning.

## Supporting information

S1 Data (XLSX)
